# Prevalence of cervical and apical root resorption in maxillary central incisors without orthodontic treatment: A cone-beam computed tomography study in Peruvian individuals

**DOI:** 10.4317/jced.63066

**Published:** 2025-09-01

**Authors:** Kevin Edgard Arana-Calderón, Raisa Soledad Sairitupac-Ayala, Luis Ernesto Arriola-Guillén

**Affiliations:** 1 Undergraduate student, School of Dentistry, Universidad Científica del Sur, Lima, Perú; 2 Ph.D. and Associate Professor of the Division of Orthodontics, Universidad Científica del Sur, Lima, Perú

## Abstract

**Background:**

This study aimed to determine the prevalence of cervical and apical root resorption in maxillary central incisors of Peruvian adults without a history of orthodontic treatment, using cone beam computed tomography (CBCT).

**Material and Methods:**

This was an observational, descriptive, and cross-sectional study. The sample included 103 CBCT scans from adult individuals in a Peruvian population, comprising 71 women and 32 men (mean age: 34 ± 32 years). DICOM files were analyzed using RayScan Expert 3D software. The presence of cervical and apical root resorption in permanent maxillary central incisors was assessed by examining coronal, sagittal, and axial sections. Demographic characteristics were also recorded. Fisher’s exact test and binary logistic regression were used to evaluate associations, with a significance level set at *p* > 0.05.

**Results:**

The overall prevalence of root resorption was 5.8%, with cervical root resorption accounting for 1.0% and apical root resorption for 4.9%. No significant association was found between sex and the presence of root resorption (*p* = 0.173). Neither sex nor age had a direct influence on the occurrence of root resorption.

**Conclusions:**

The prevalence of cervical and apical root resorption in maxillary central incisors of untreated Peruvian adults was low. Nevertheless, this condition should be considered by orthodontists during diagnosis and treatment planning.

** Key words:**Cone-Beam Computed Tomography, Incisor, Root Resorption.

## Introduction

External root resorption is an unexpected process characterized by the loss of dental hard tissues, specifically cementum and, in some cases, dentin, due to odontoclastic activity [1]. In primary teeth, root resorption is usually physiological and desirable, as it facilitates the eruption of the permanent successor tooth. In permanent teeth, however, root resorption may occur either within the root canal or on the external surface of the root. In advanced cases, the defect can progress toward the crown, potentially leading to the loss of the affected tooth. The process is typically asymptomatic and irreversible [1-5].

The causes and mechanisms underlying root resorption are not yet fully understood. However, several factors have been identified as potential causes, including trauma, pulp infection, tooth whitening, and orthodontic treatment that involve prolonged and excessive forces [4]. This condition can be detected only through radiographic or tomographic examinations. Cases have also been reported in individuals who did not undergo orthodontic treatment, emphasizing the need for increased awareness when planning orthodontic procedures [1]. By understanding these risk factors, we can identify the onset of resorption and determine its stage, which is crucial for preventing further progression of the condition.

Currently, cone-beam computed tomography (CBCT) is widely used in dentistry. In cases of apical or cervical root resorption, it enables more accurate evaluation by providing a clear view of the tooth without overlapping structures or distortions. This allows for detailed visualization of the resorptive defect and accurate staging, helping the clinician develop an appropriate treatment plan [2]. To date, studies have primarily focused on evaluating apical rather than cervical root resorption in patients prior to undergoing orthodontic treatment. Reported prevalence rates ranged from 0.08% to 2.3% [1]. However, these studies did not provide clear information regarding cervical resorption in the evaluated teeth. Understanding its prevalence is important to determine the baseline condition of patients prior to initiating orthodontic treatment. By being aware of these percentages, orthodontists can take them into account when planning their treatments, especially since this type of resorption has been reported to increase following orthodontic procedures [1]. Therefore, this study aimed to determine the prevalence of cervical and apical root resorption in maxillary central incisors of Peruvian individuals who had not undergone orthodontic treatment, using CBCT.

## Material and Methods

The study followed an observational, descriptive, and cross-sectional design. The Institutional Research Ethics Committee of Universidad Científica del Sur approved the study under code 933-CIEI-CIENTÍFICA-2025. The study ensured the protection of participants’ rights, safety, and well-being.

The study population consisted of cone-beam computed tomography (CBCT) scans retrieved from the radiology department database of Universidad Científica del Sur. These scans belonged to individuals who visited the facility for reasons unrelated to the study but were seeking orthodontic treatment between March 2, 2023, and July 30, 2024. A representative sample of 103 CBCT scans was determined using the formula for estimating a proportion (specifically, the proportion of cervical resorption in the evaluated scans), based on a previously reported prevalence of approximately 2.3% [1]. The sample size was calculated with a confidence level of 95%, statistical power of 80%, and a precision of 5%. The calculation was performed using the software available at Fisterra.com.

Inclusion criteria consisted of CBCT scans obtained at the radiology center of Universidad Científica del Sur from both male and female individuals, with high contrast resolution and image clarity without a history of orthodontic treatment. Exclusion criteria included CBCT scans showing cracks near the area of interest, neoplasms, lesions, dental anomalies, or any other abnormal findings.

**Figure 1:**
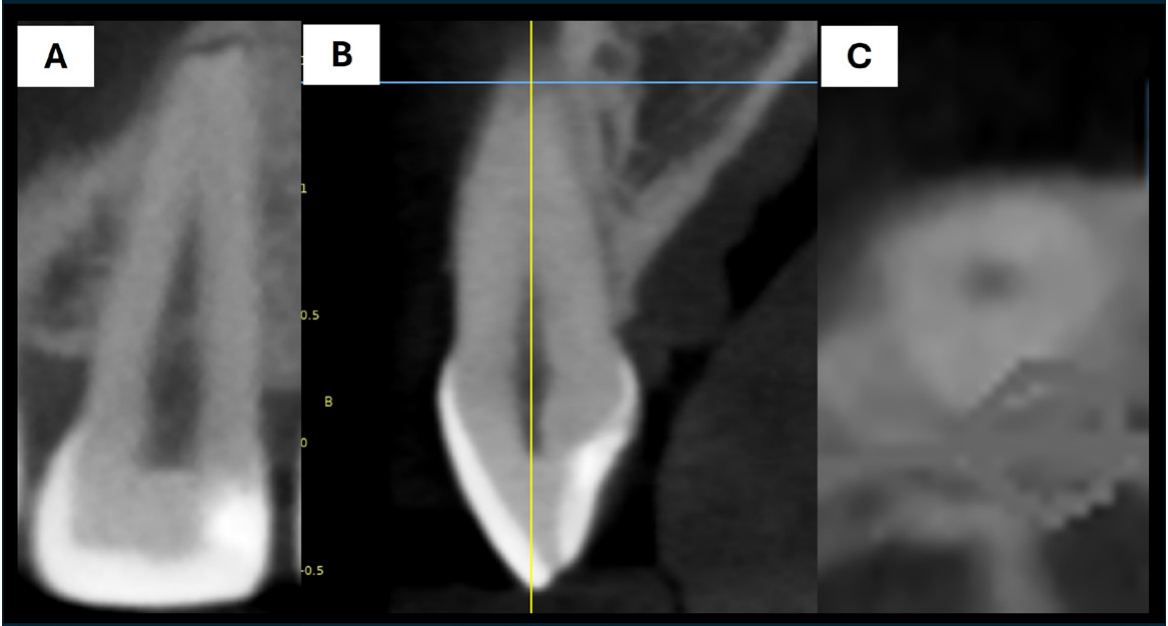
Measurements of an upper incisor exhibiting root resorption. (A) Coronal section. (B) Sagittal section. (C) Axial section.

The CBCT scans were obtained using a RayScan Expert 3D cone-beam computed tomography scanner (Terarecon, San Mateo, CA, USA), following a high-resolution protocol (90 kVp, 4 mA, field of view [FOV] 5 × 5 cm, voxel size of 0.1 mm, and 360° rotation). The CBCT images were processed using RadiAnt DICOM viewer (Medixant, Poznan, Poland) and assessed in a dimly lit room. The evaluation was conducted using multiplanar reconstruction (MPR), with the use of image enhancement tools such as brightness, contrast, and zoom. The computer used to view the CBCT images was an Asus VivoBook X415EA 11th Gen laptop with 8 GB RAM and 475 GB of storage. Cervical and apical resorption were evaluated using sagittal, coronal, and axial sections of the CBCT scans, enhancing image sharpness and angulation to obtain a clearer view. The diagnosis of root resorption required identifying the loss of continuity of the root surface, along with irregularities in the root contour at the cervical or apical level (Fig. 1). Resorption was classified as either absent or present, and the type of resorption was categorized as cervical, apical, or both.

A researcher responsible for the measurements was trained and calibrated by an experienced orthodontist with over ten years of experience to determine the presence of root resorption. The Kappa statistic was used to assess inter-operator agreement, yielding a value greater than 0.8 in all evaluations.

- Statistical analysis

SPSS version 19 for Windows (IBM, NY, USA) was used. Associations between the presence of cervical or apical resorption and covariates were assessed using Fisher’s exact test, and binary logistic regression was subsequently used to determine the influence of predictor variables (sex and age) on the occurrence of root resorption. The significance level was set at *p* > 0.05.

## Results

 Table 1 presents the baseline characteristics of the sample.  Table 2 shows the prevalence of root resorption at both the apical and cervical levels, with an overall prevalence of 5.8%, 4.9% at the apical level, and 1.0% at the cervical level. No significant association was found between sex and the presence of root resorption (*p* = 0.173, Fisher’s test) ( Table 3). When evaluating whether the assessed covariates—sex and age—had a direct influence on the occurrence of root resorption, no significant effect of these variables was found overall (*p* < 0.05) ( Table 4).

## Discussion

Root resorption is a process frequently observed during orthodontic treatment [27]. It is characterized by the loss of dental hard tissue, i.e., cementum and sometimes root dentin, due to odontoclastic activity [27]. The etiology and pathogenesis of root resorption are not fully understood, and the most identified causative factors include trauma, pulp infection, and orthodontic treatment involving excessive forces [5]. However, its presence in lower percentages prior to orthodontic treatment has been reported in a few previous studies [1]. One study found a similar prevalence using cone-beam computed tomography (CBCT), suggesting that such lesions may also occur in patients without previous interventions. Nevertheless, cervical-level resorption has been reported far less frequently [5]. Most of these studies focus primarily on the progression of apical defects, leaving a significant gap in the understanding of cervical involvement. For these reasons, this study aimed to determine the prevalence of cervical and apical root resorption in the maxillary central incisors of adult Peruvian individuals without a history of orthodontic treatment.

In our study, the overall prevalence of root resorption among the evaluated patients was 5.8%. These results confirm that it is an uncommon condition, but it should not be overlooked [1]. Although cases of root resorption are rare, their identification is crucial, as many instances do not have an apparent cause. These cases can be classified as idiopathic root resorption, meaning they are not directly linked to known factors such as trauma, infections, or previous orthodontic treatment [6]. Notably, the root resorption rates observed in our study were similar to those reported by Ferreira *et al*. [1], who found a prevalence of 1.35%. This Figure is significantly lower than the prevalence observed in our study.

The diagnostic evaluation of the patient is essential for ruling out this lesion, as neglecting to consider it may lead to complications during orthodontic treatment. This study aids clinicians by providing a clearer understanding of the prevalence of root resorption, which should be considered when planning treatment. Additionally, it highlights the importance of using appropriate tomographic evaluations in these cases [4].

Among the types of resorptions detected, apical resorption was the most frequent (4.9%), while cervical resorption was the least common (1.0%). This predominance of apical resorption is consistent with previous studies, such as those by Dao *et al*. [6], which suggest that this localization may be due to biomechanical factors or even individual predisposition. However, in this case, the patients evaluated had no history of orthodontic treatment, which reinforces the hypothesis of an idiopathic etiology [6,9].

In our study, resorption cases were found only in women, and no cases were detected in men; however, this difference was not statistically significant (*p* = 0.816). Similarly, logistic regression analysis indicated that neither sex nor age was significantly associated with the occurrence of root resorption. This suggests that the condition may be related to other variables not evaluated in this study, and future research could expand on this line of investigation. An important aspect of this study was that the diagnosis of root resorption was based on a clearly visible disruption of the root surface continuity and surface irregularities at both the apical and cervical levels. For this reason, there was no possibility of confusion or diagnostic error with conditions such as root dwarfism or other periapical pathologies [9,10,26].

Finally, dentists and orthodontists must be aware of this condition before initiating orthodontic treatment. Early detection through imaging studies, such as tomography, facilitates better collaboration among professionals. This approach enables more careful and personalized clinical management, reducing the risk of exacerbating these defects through the application of orthodontic forces [27].

## Conclusions

The prevalence of cervical and apical root resorption in maxillary central incisors of adult Peruvian individuals without a history of orthodontic treatment is low. Nevertheless, this condition should be considered by dentists—and even more so by orthodontists—since, if present, the application of orthodontic forces could exacerbate the lesion.

## Figures and Tables

**Table 1:** Initial characteristics of the sample.

Sex	n	Mean	S.D.
Male	32	31.84	8.98
Female	71	33.63	9.36

*p* = 0.365 Student’s t-test

**Table 2:** Prevalence of root resorption (apical and cervical) in the evaluated CBCT scans.

Root resorption	n	%
Absent	97	94.2
Present	6	5.8
Total	103	100.0
Root resorption by type		
Apical	5	4.9
Cervical	1	1.0
Absent	97	5.8
Total	103	100.0

**Table 3:** Association between root resorption and sex.

Sex		Root Resorption		
		Absent	Present	Total
Male	n	32	0	32
	%	100.0%	0.0%	100%
Female	n	65	6	71
	%	91.5%	8.5%	100.0%
Total	n	97	6	103
	%	94.2%	5.8%	100.0%

*p* = 0.173, Fisher’s exact test

**Table 4:** Binary logistic regression assessing root resorption based on predictive variables.

Variable	p	Exp(B)	95% C.I. for Exp(B)	
			Lower	Upper
Sex (Male)	-	-	-	-
Sex (Female)	0.998	138233437.1	0.000	
Age	0.409	1.038	0.950	1.135
Intercept	0.997	0.000		

## Data Availability

The datasets used and/or analyzed during the current study are available from the corresponding author.
